# Reduction of the Femur without Pulleys or Any Other Mechanical Means

**Published:** 1852-02

**Authors:** 


"*Reduction of the Femur without Pulleys or any other mechanical means.*"

To the Editor of the Boston Medical and Surgical Journal.

Dear Sir, — Since you did me the favor to republish, in your Journal, my article on the reduction of the femur without pulleys or other mechanical means, I have been honored with several notices and strictures through the same channel. Foreseeing something of the kind, and not wishing to be drawn into a personal controversy, I had proposed, from the beginning, not to reply to any such criticisms. And in deviating *now* from my first resolve, I do so, more for the sake of establishing *certain facts and principles,* which I deem important, and of correcting some misapprehensions of my critics, than for the sake of controversy.

I am accused, either in direct terms or by implication, of making statements that are untrue, of setting up a claim to originality, and of appropriating to myself the honor that justly belongs to others.

Dr. Cartwright informs us that he "first reclaimed from the chaos of blundering empiricism, and brought under the empire of the laws of science," this old method of reducing the femur; that "after long pondering over the cases of dislocation reduced by accident, the truth flashed into his mind, that force had nothing to do with it" — and that as long ago as in May, 1844, he "composed and published an elaborate essay *to prove its practicability*, by quoting cases recorded in standard works on surgery." "Unlike Dr. Reid, he did not quote his own cases reduced by *the method advised*," &c.

Now as I commenced my investigations in 1828, and pursued them at intervals till 1839, and as my first case occurred in March or April of 1844, one or two months before his publication; and as I have never had the pleasure of seeing his essay, it is manifest I am not guilty of purloining anything from his labors; but, it would seem, guilty only of doing what he himself had done, viz., daring to observe, think and act for myself without authority, at or about the same period of time.

But I — very improperly, as he would intimate — quoted my own cases; and why? Because I wished "to prove" — not a *probability* — not a speculation *to be practicable* — but to establish *facts and principles.* To do this I preferred to give observations, experiments, operations and cases — not *accidental ones,* described in loose and general terms, whose history and description were necessarily defective; but cases, for whose history and description I could vouch as correct in every particular; and operations, undertaken, conducted and completed on the *principles* sought to be established, and which operations were, therefore, not *accidents*, but *legitimate results of the principles adduced.* In this way I hoped, by giving my own observations and experience, to contribute my mite to the general stock of surgical knowledge. If, in all this, there were anything improper, immodest, or egotistical, why then I simply plead guilty, and patiently await my sentence.

Then, again, other parties are aggrieved, and we are told, with some apparent degree of sensitiveness, that my "claim to originality for a new method of practice will fall upon *a large class* of your New England readers as a very old and familiar plan; that this so-called original method was familiar to *many* of our profession;" that I had said, "that this method was contradictory to the teachings of all standard writers on surgery," and had enumerated several of the principal American and English standard authors, who uniformly taught a different method, the pulley system—but did not include in the list of standard authors on surgery the names of Professors Nathan and N. R. Smith. For this omission and other delinquencies the reviewers call me to account. One says, "We will immediately show that it is *not* contradictory to the teachings of all standard writers on surgery;" and asks why I omitted in my list the name of Nathan Smith. He then refers us to "Smith's Medical and Surgical Memoirs," published in 1831, by Prof. N. R. Smith of Baltimore, from which he quotes several paragraphs, and likewise from my essay, and "thinks if any one will take the trouble to compare these extracts he cannot fail to see that they describe, *in substance*, one and the same thing." To all this I wish to say—

*First.* That if only "a large class" of New England physicians and surgeons—if only "many of our physicians," were familiar with this method, and not the whole, both in this country and Europe, and wherever the *unscientific* and cruel method of *traction* by pulleys is taught and practiced, I hope I have done the profession at large no disservice.

*Second.* Far be it from me to detract one iota from the fame and just dues of Prof. Nathan Smith. I yield to no man in admiration and esteem for his genius, talents and skill, although I never had the honor of his acquaintance or of listening to his lectures. His reputation constitutes an important part of the glory of American surgery, and is therefore the common property of the profession.

*Third.* According to my notion, "a standard work on surgery" is one embracing the whole subject and embodying the received and acknowledged principles and practices of the profession. This is not the character of "Smith's Medical and Surgical Memoirs." The work, as its title imports, is a miscellaneous collection of several subjects. One article only treats of dislocation and reduction of the femur, and that is from the pen of Prof. N. R. Smith. And this, he tells us, is not intended as a "complete treatise on these dislocations;" for such, he "refers us to systematic works." This is designed, as he informs us, "to be supplementary to the information which we now possess, and to subvert certain erroneous practices." These were exactly my objects; to contribute—not to collate and compile—and likewise "to subvert," if possible, "certain erroneous practices."

Again, Professor Nathan Smith's theory and practice, as taught by himself, or as presented in his "Memoirs" by Prof. N. R. Smith, have never, to my knowledge, found their way into any standard surgical work, nor even been noticed by any author. A few or many of his pupils may have adopted them as authority. But the profession as a body have never received and acknowledged them, either theoretically or practically. Besides, the theory and practice inculcated, are but partially true. These are the reasons why I did not include said Memoirs among the standard works on surgery.

But I did not intentionally do either Prof. N. Smith or Prof. N. R. Smith injustice.

In my address, as originally delivered and first published in the Buffalo Journal, I said, "I was aware that Prof. Nathan Smith, of New Haven, had, in his day, taught in his lectures a somewhat similar method—perhaps the same; but none of his pupils whom I had ever met, could describe either his method or the rationale of it. I had seen, too, his Memoirs, published by his son, Professor N. R. Smith, of Baltimore, but he confesses he did not recollect the teachings of his own father, and that he, the elder Smith, had left no notes or records of his doctrines or practice." [In this sentence I have done injustice to Prof. N. R. Smith. When this paragraph was penned I had not a copy of his Memoirs before me. I had never seen but one copy, in 1838, which was after I had made the chief part of my observations. He says, "The principles which I shall endeavor to establish, were derived in part from my father's lectures" — and just after, when referring to *the case* which he *used* to relate in his lectures, he says — "Notes of *this case*, unfortunately, I am not able to discover among his papers." And so of a letter, addressed to his father by a medical gentleman who had *a* similar case, "it could not be found." The impression left upon my mind by these statements, led to the above error. This correction and acknowledgment are therefore due to Prof. N. R. Smith.] "Dr. N. R. Smith, however, proceeds to give what seems to him the probable doctrines inculcated by his father, and gives directions for reducing dislocations of the hip, with drawings illustrative of his method. But it is apparent, that when he wrote his book and gave these directions and illustrations he had never reduced a hip by his method. For his directions require impossibilities, and his illustrations (drawings) are mere fancy; no such thing in nature can exist. For to *abduct* a thigh dislocated on the dorsum of the ilium, *before* flexing it on the pelvis, or to *abduct* and *flex* at the same time, *as he directs*, is absolutely impossible, without rupturing the obturator externus — and to rupture this in order to obtain flexure, would require the power of many men; but to flex the leg first on the thigh, *then adduct* the thigh, carrying it even *over the sound one, and at the same time flex the thigh on the pelvis, carrying the knee over and upward by a kind of semi-circular sweep, is a very different and a very easy thing.*" And I may here add my honest conviction, that if any one ever did succeed in reducing the hip, by attempting to observe the directions given by Dr. N. R. Smith, he did it by "making experimental bending movements of the limb," in *every possible direction*, as advised by Dr. Dorsey, and quoted by Dr. Smith, and thus by accident *adducted and flexed the thigh on the pelvis;* performing empirically and by *accident*, the *adducting* movement, which I have demonstrated that both the anatomy and mechanism of the parts require.

By the foregoing quotation it will be seen, that I gave to Prof. Nathan Smith, credit for all that I then knew of his teachings and practice; and therefore, although in discussing the subject, I called it "my method," I set up no claim to priority or originality over him for the *general mode* of operating. What I claim is, the discovery of the *true principles and rationale* of the operation, and the *specific movements* required by these principles. Whether Prof. N. Smith fully understood *these principles*, is uncertain. That Prof. N. R. Smith did not, appears from the whole tenor of his treatise. And until the recent attempts to throw light on the subject, I was inclined to believe that Dr. N. Smith understood them better than was represented in his Memoirs; but these attempts have served to render it probable that Prof. N. R. Smith has given us the sum total of all that was known to both.

He says, "The principles which I shall endeavor to establish were derived *in part* from my father's lectures." The first and principal portion of the essay is a hypothetical argument to show that muscles are themselves the chief agent in producing dislocations, and that therefore we might, "if we knew how," make them subservient to the reduction. The last part of the essay contains his "*proposed method of effecting* the reduction of the os femoris." Not knowing what *part* belongs to the elder, and what to the junior Smith, we must take it as a whole, and as embodying all that was known to both; and what was that? A few quotations only can be given in an article like this; the reader is referred to the Memoirs themselves, where he will find the *principle* relied upon, is, that the *powerful contraction of muscles* which dislocated the bone, must be employed to reduce it; that the leg is to be used as a long lever by which "*to multiply force*," to call certain muscles into action, and by "adroitly" "making bending movements" we are "to evade" the resistance of the powerful muscles—for when "we endeavor to effect the reduction of the bone by extension made by pulleys, the extending effort which we then make *is directly resisted by the glutei muscles*."

In the Memoirs, we are told, that "Prof. Nathan Smith *used to relate*, in his surgical lectures, *a case* of dislocation of the os femoris on the dorsum ilii, in which he promptly succeeded, by the mere force of his hands, in effecting the reduction. Notes of *this case*, unfortunately, I am not able to discover among his papers." * * * "*After attempting* the ordinary methods, by extension, in vain, he bent the leg on the knee, seized the leg, and *used it as a lever, rotated the thigh a little outward*, then gently ABDUCTED the thigh, and lastly flexed it freely on the pelvis, by carrying the knee toward the face of the patient. * * * A medical gentleman of Massachusetts saw *a* similar case of dislocation, practiced the same method, and succeeded with equal facility. A letter from him to Professor Smith, detailing the particulars of the case, I once saw, but unfortunately it cannot now be found." Again, after referring to the case which occurred in the hands of Dr. Physick, he says, "*The case in which my father succeeded*, was one of dislocation on the dorsum illi."

Now several things here are worthy of notice. 1. It is "*a case*," "*the case*," which he "*used to relate* in his surgical lectures," leaving us to infer that he had no other case of his own *to relate;* if he had any other, why refer to the *one other case* of the gentleman of Massachusetts? 2. He succeeded in *this case*, "*after attempting the ordinary methods of extension in vain*." From this, it is fair to infer that *this case* was somewhat *accidental*, and it was *the case* on which he founded his subsequent teachings—or, that he had so little confidence in his new method, that he was fain to try the ordinary modes first. 3. Prof. N. R. Smith, the son of Prof. Nathan Smith, who must have enjoyed both the private and public instructions of his father, had access to and control of all his papers, and from which he compiled his Memoirs, is able to quote but *one single case*, occurring in his father's practice, and that of a somewhat doubtful or *accidental* character. And what have "*Suum Cuique*," and Drs. J. M. Smith and M. F. Colby done? Manifestly, all have quoted *this same case* to establish the claims of Dr. N. Smith.

Now for the *rationale* of the operation. He refers to "a case which fell under his [Dr. N. R. Smith's] own observation," in which "the most powerful and persevering efforts had been made by the the aid of pulleys, but without success—made, too, by men of *science and skill;* the case was then dismissed as unmanagable. In a few hours afterward the patient fell into the hands of a quack—who, without any assistance, moved the knee in *various directions*" and reduced the bone. After quoting the case of Mr. Cornish, recorded by Sir A. Cooper, and the case of Dr. Physick, recorded by Dr. Dorsey—after quoting the recommendation of the latter, viz., "*to try every possible motion of the limb*, before abandoning the case as hopeless, as, very often, after force has failed, a gentle effort in some *new* direction is found successful," he then says — "But if these gentle efforts, these *experimental bending movements* of the limb, so often succeed in the worst cases, and after the most powerful efforts have been made in vain, does it not go far to prove, that gentle means, if *adroitly* employed, would succeed better in all cases? There is no doubt a *constant mechanical principle upon which the reduction is effected* in such cases, and one which would *perhaps* succeed in *nearly* all cases, *if we knew how to employ it understandingly and with precision*, and did not avail ourselves of it by *mere hap-hazard*. If a gentle movement of a peculiar kind succeed in *one case* of complete dislocation on the dorsum ilii, after all other means have failed, ought not *this movement, if well understood*, to succeed in other cases better than the usual mode? The *mechanism* of these dislocations is certainly the same in all this variety — the bone assumes the same attitude, and the muscles assume the same relations — furnishing the same impediments and the same aids in every case. This frequent *failure of art* and the *success of accident* satisfy me, that there is some *important principle* relative to the mechanism of this dislocation WHICH IS NOT YET UNDERSTOOD." Again, after describing his "proposed method," he says, "The cases, in which lateral movements with gentle force have succeeded, either by design or fortuitously, * * * induce me *to believe* that there is *a secret* method in which we may uniformly succeed; and that method *I believe* to be the one in which the movements described above are employed."

Here, then, all is conjecture—conjectures, valuable, important, and approximating to the truth. One fact only was certainly known, viz., that reductions had been effected "by lateral bending movements," whether made "by design or fortuitously;" and on these conjectures, embracing the general truth, to be sure, but yet the specific items unknown, he proceeds to give a "proposed method." Whereas, I think I have discovered and demonstrated the cause of "*these frequent failures of art*;" "*the important principle*, relative to the *mechanism* of these dislocations, which has [not] heretofore been ununderstood;" "*the secret method;* the [true] bending movements to be made"—*not hap-hazard and adroitly*—not indefinitely "in every possible" way — but in a certain order, step by step, which never fails to secure the desired result.

In his "proposed method," he directs the patient to be laid on his sound side: I place him on his back. He lashes him fast to the table — and then "the operator designs to employ any degree of extension," and must put also a counter-extending band over the perineum: I use no fixtures of any kind. He next flexes the leg on the thigh: in this we agree. He *then rotates* the limb: this is useless, and serves only to give pain to the patient. He next *gently abducts* the thigh: this is worse than useless, as it increases the *tension of the obturator externus*, already *strained* to its utmost; unnecessarily tortures the patient, and is incompatible with the next movement. Instead, therefore, of "*gently abducting*" the thigh, I *strongly adduct it*, and thereby relax the stretched muscles. He next flexes the thigh on the pelvis, *increasing abduction:* I flex on the pelvis, merely *continuing adduction*, and do not *abduct* nor *rotate*, till the knee is as high as the umbilicus. These differences in our mode of operating will be found to be important. My method is more simple; requires less time; is easier, requiring less force; causes less pain, and is in exact accordance with the mechanism of the dislocated joint.

Without disparagement, therefore, of the Professors Smiths or any one else, I believe I may justly speak of the mode which I have described as "my method."

1. Because, my observations and *experiments* were made without the aid or first suggestion of any one, and before I had seen Smith's Memoirs.

2. Because, so far as I know, I first discovered and demonstrated that the *contraction and resistance of the large muscles do not constitute the impediments* to be overcome, as has been taught by all surgical writers, not excepting even Prof. N. R. Smith; but,

3. That the real and almost the whole difficulty lies in the *distension* of the comparatively small muscles, viz., the obturator exturnus and internus, the pyriformis, gemelli, and quadratus.

4. I have proved, by actual experiment, that muscles are incapable of extension beyond their normal length, to any practicable degree, without danger of laceration.

5. I have demonstrated *mathematically* that *traction* on the shaft of the bone, by pulleys or other means, is incompatible with the mechanical and physiological action of the muscles, impeding the reduction; that it increases the difficulty, tends to rupture the aforesaid muscles, cruelly and unmercifully tortures the patient, and is therefore an *unscientific* application of force.

6. And, lastly, I have pointed out the *true and only evolutions* of the limb, required by the mechanism of the joint in order to reduce, without mechanical power, this particular and heretofore formidable dislocation.

*Rochester, N. Y., Dec.* 12, 1851. W. W. Reid, M. D.

